# Neck complaints before and after uncomplicated thyroidectomy: prevalence, postoperative outcome and relationships with thyroid weight and reflux like symptoms

**DOI:** 10.1007/s12020-020-02568-y

**Published:** 2021-01-06

**Authors:** Maria Raffaella Marchese, Jacopo Galli, Lucia D’Alatri, Annamaria D’Amore, Francesco Sionne, Lucrezia Trozzi, Gaetano Paludetti, Rocco Bellantone, Celestino Pio Lombardi

**Affiliations:** 1 Department of Aging, Neuroscience, Orthopedics and Head and Neck Sciences, UOC di Otorinolaringoiatria, “Fondazione Policlinico Universitario A. Gemelli IRCCS”, Rome, Italy; 2 Università Cattolica del Sacro Cuore, Rome, Italy; 3 Department of Gastroenterologic, Endocrine-Metabolic and Nephro-Urologic Sciences, Division of Endocrine Surgery, “Fondazione Policlinico Universitario A. Gemelli IRCCS”, Rome, Italy

**Keywords:** Thyroid, Thyroidectomy, Voice, Swallowing, Dysphagia, Dysphonia, Reflux disease, Thyroid weight

## Abstract

**Purpose:**

The surgical thyroid disease includes upper aerodigestive complaints with not homogenous prevalence and specific features. The purpose was to analyze before and after total thyroidectomy (TT) the prevalence and severity of voice, swallowing, respiratory, and reflux airway symptoms in relation with thyroid weight.

**Methods:**

A total of 98 consenting patients undergoing TT were enrolled. Preoperatively, 1 and 3 months after TT, patients underwent videolaryngoscopy, subjective evaluation of voice (VIS), swallowing (SIS and EAT-10), respiratory (mMRC), and reflux symptoms (RSI, Gerd-Q). The scores were analyzed based on thyroid weight (<25 gr, 26–50 gr, 51–75 gr, >75 gr) and post-operative score gain was calculated from the score before TT and the follow-up examination.

**Results:**

In total, 40/98 selected cases of uncomplicated TT completed the postoperative evaluation. Endoscopic signs suggestive of reflux disease were observed in 1/40 (2.5%) and 0/19 cases before and after TT respectively. The prevalence of cases with abnormal reflux symptom index decreased significantly after surgery (8/40 vs 1/40) (*p* < 0.05), similarly occurred for the Gerd-Q (4/40 vs 1/40) (*p* < 0.05). Three months after TT the voice, swallowing and respiratory scores were significantly lower than the preoperative ones (*p* < 0.05). The SIS correlated positively with EAT-10 and RSI. After 3 months the postoperative score gain of voice, swallowing, respiratory, and reflux symptoms (Gerd-Q) was statistically higher (*p* < 0.05) in the cases with heaviest gland.

**Conclusions:**

The surgical thyroid disease is associated to mild aerodigestive preoperative compressive symptoms, that include respiratory abnormalities and reflux like symptoms, regardless of the gland weight. In absence of endoscopic signs of airway reflux the presence of reflux symptoms suggests an overlapping with thyroid neck complaints. The patients undergoing uncomplicated TT had improvement in compressive symptoms and the greatest improvement is seen in larger goiters.

## Introduction

Swallowing, voice and throat discomforts are common findings in patients with thyroid nodules and they can appear or persist after thyroidectomy even in the absence of laryngeal nerve injuries and videostrobolaryngoscopy (VLS) alterations [1–7]. In two large studies compressive symptoms ranged between 11 and 22% of patients [8, 9] even if higher percentages up to 52% were recently reported [10, 11]. Compressive symptoms are usually correlated to the mechanical effects of nodular goiter. On the other hand, the prevalence of voice complaints after uncomplicated thyroidectomy varied between 25–90% and between 11–15% for early and persistent disturbances, respectively [7]. Postoperative symptoms are primarily related to a supposed injury to the perivisceral neural plexus innervating the pharyngeal and laryngeal structures [12] or to a normal healing process presumed to result in laryngotracheal fixation with impairment of vertical movements [3, 13]. Recently some authors hypothesized that pre- and post-operative local neck symptoms may be related to reflux laryngitis [14, 15]. However, to clarify the role and the relationship between thyroid disease and postsurgical neck complaints it is of paramount importance to define the symptoms in terms of percentages and severity by resorting validated methods and to review the outcome of uncomplicated thyroidectomy. The purpose of this research was to study the prevalence and severity of reflux symptoms and respiratory abnormalities in addition to voice and swallowing complaints before and after thyroidectomy and the relationships between the subjective assessment of reflux and dysphagia. In addition, data was also collected to provide our contribution to understand the functional impact of thyroid weight before and after thyroidectomy.

## Materials and methods

Patients scheduled to undergo total thyroidectomy (TT), from January 2018 to July 2019, were considered eligible for this study. The indication for surgery was toxic goiter, THY3 (sec. Bathesda system; TIR3B sec. SIAPEC consensus), symptomatic multinodular goiter. Exclusion criteria were age <21 and >65 years, previous vocal fold paralysis, gastroesophageal reflux, use of PPI, H2 blocker or other reflux therapy, BMI >30, voice users, smokers/former smokers, history of voice, or laryngeal disease requiring therapy or of pulmonary disease, previous neck surgery, malignancy other than papillary thyroid carcinoma. Patients showing signs of inferior laryngeal nerve (ILN) after thyroidectomy or external branch of superior laryngeal nerve (EBSLN) palsy at VLS examination were excluded from the study. Based on the histopathological findings, the thyroid weight was stratified as follows: <25 gr (group I), 26–50 gr (group II), 51–75 gr (group III), >75 gr (group IV). A written informed consent was obtained from all the participants included in the study.

### Surgical technique

An experienced endocrine surgeon (CPL) performed all surgical procedures. TT is defined as total bilateral extracapsular thyroidectomy. Typically, after skin incision, the strap muscles are separated along the midline and then reapproximated at the end of the operation. In every thyroid surgery, a systematic and prompt identification of the ILN is recommended. Following its identification, the surgeon tracks the ILN upward to the point of entry into the larynx. Every attempt should be made to preserve all ILN branches during dissection. If the nerve is not found at its usual place, the surgeon should be aware of the possibility of a nonrecurrent ILN. Because the EBSLN is not exposed routinely, the superior thyroid artery and vein are ligated individually, close to the thyroid capsule to avoid injury to the EBSLN. When the EBSLN is not readily identifiable, no further dissection is pursued to avoid inadvertent nerve injury. In addition, it is important to avoid producing lesions of the cricothyroid muscle from electrocoagulation or manual retraction.

### Videostrobolaryngoscopy

The VLS was performed by an expert, independent and blinded ENT doctor (LD, MRM), using a flexible laryngoscope (model Xion Gmbh Video-Nasopharhingoscope EV-NE) to assess vocal folds motion impairment (ILN injury) as well as laryngeal signs of laryngopharyngeal reflux (LPR) according to Belafsky’s Reflux Finding Score (RFS) (range from 0 to 24) (cut-off = 7) [16].

### Subjective voice, respiratory, and swallowing evaluation

Patients were asked a series of items related to the frequency of voice and swallowing abnormalities and to the dyspnea degree using three different questionnaires. For the voice assessment we administered the voice impairment score (VIS) questionnaire specifically developed by us in the past for thyroidectomized patients and then widely used [3, 13, 17]; VIS ranges from a minimum score of 0 (no voice alterations) to a maximum of 40 (greatest voice impairment). For respiratory assessment we administered the modified British Medical Research Council Questionnaire (mMRC), which is a self-rating tool to measure the degree of disability that breathlessness poses on day-to-day activities on a scale from 0 to 4. Finally, the subjective swallowing quality was assessed by administering two questionnaires: Swallowing Impairment Score (SIS), with scores ranging from 0 (no swallowing alterations) to 24 [17] and the Eating Assessment Tool (EAT-10) [18]. The EAT-10 is well known in the literature as a validated test to document the dysphagia severity and monitor the treatment response (total score ranges from 0 to 40; score of 3 or higher is abnormal).

### Reflux symptoms questionnaires

To detect symptoms suggestive for reflux, we administered to all patients Reflux Symptom Index (RSI) [19], which includes 9-items, as an instrument for LPR (cutoff = 13) and Gerd-Q, which is a recently developed 6-item questionnaire, as a tool to improve and standardize symptom-based diagnosis and evaluation of treatment response in patients with GERD (cutoff score = 8) [20].

Post-operative score gain was calculated from the score before the operation and the follow-up examination.

In order to obtain an outcome more clinically oriented, we divided the distribution of all questionnaire scores into the following classes: SIS 0 (no dysphagia), 1–6 (mild), 7–12 (moderate), 13–18 (severe), 19–24 (very severe); EAT-10 0–3 (no dysphagia), 4–10 (mild), 11–20 (moderate), 21–40 (severe); VIS 0 (no dysphonia), 1–10 (mild), 11–20 (moderate), 21–30 (severe), 31–40 (very severe).

### Patients’ assessment and timing of evaluation

All patients underwent VLS and self-assessment questionnaires to evaluate symptoms related to reflux disease and voice, swallowing and respiratory quality. The instrumental and subjective assessments were performed before TT and 1 and 3 months after surgery.

### Statistical analysis

Statistical analysis was performed using a commercially available software (Excel – Microsoft Corporation, Redmond, Washington, USA). Continuously distributed outcomes were summarized as mean and categorical outcomes with frequencies and percentages. Pearson and Spearman correlations was used to indicate the statistical relationship between the scores of questionnaires used. The analysis of variance (ANOVA) for repeated measures was used for continuous variables. The Wilcoxon signed rank test and Student *t* test were used for nonparametric and parametric variables respectively, when appropriate. The *χ*^2^ test was used for categorical variables. The significance level was set at 0.05.

## Results

Among the 98 cases who met inclusion criteria, 1 (1.02%) patient experienced transient laryngeal nerve palsy, documented by postoperative FFL, and was excluded from this study. Among the remaining patients, 57/97 (58.76%) refused to undergo a follow-up consultation because of the absence of symptoms 1 month after the operation (3/57–5.26%) or because they lived far from the hospital (54/57–94.72%). Furthermore, 40/57 (41.24%) patients completed the long-term follow-up procedure and accepted participation in the study. The female were 36/40 (90%) and the males 4/40 (10%) with a mean age of 53.15 years (range 22–65 years). The final histologic examination of the resected tissue confirmed benign diseases in 35/40 (87.5%) patients and thyroid carcinoma in 5/40 (12.5%) cases. The mean weight of the surgical specimens was 42.56 g (range, 11.6–139 g). The percentages of cases divided by the thyroid weight groups were 15/40 (37.5%) for group I, 12/40 (30%) for group II, 6/40 (15%) for group III, 7/40 (17.5%) for group IV.

### Videostrobolaryngoscopy

According to the VLS data, none of the cases demonstrated any vocal fold movement limitation and/or signs of palsy of the EBSLN either before operation or postoperatively. Pre-operatively the mean RFS was 1.7 (min. 0 max. 8), 1/40 (0.4%) patient passed the cutoff (score = 8). After surgery 0/40 cases had RFS > 7. The mean post-surgery RFS were 3 (min.0–max. 6) and 4 (min. 0–max. 5) at the early and the late post-surgery control respectively. Mean postoperative and pre-operative RFS scores did not statistically significant differ (*p* > 0.05).

### Subjective voice, respiratory and swallowing results

The mean pre- and post-operative results of VIS, mMRC, SIS, and EAT-10 are reported in Table 1. One month after thyroidectomy all scores improved except for VIS. Particularly, mMRC and EAT-10 scores improved significantly (*p* < 0.05), while the VIS increase was not statistically significant (*p* > 0.05). All mean scores obtained 3 months after TT were statistically better than the preoperative scores (*p* < 0.05). The preoperative SIS showed a significant moderate positive correlation with the EAT-10 score (*r* = 0.57, *p* < 0.05). Similar correlation was observed also 1 month after surgery (*r* = 0.516, *p* < 0.05). Three months later the correlation decreased (*r* = 0.27, *p* > 0.05).

**Table 1:** Mean values of score of each questionnaire at the different time points during the study

	Pre-operative mean (± SD)	1 month PO mean (± SD)	3 months PO mean (± SD)
VIS	6.70 (±7.94)	8.97 (±8.97)	4.37 (±5.96)*
mMRC	1.33 (±1.36)	0.25 (±0.71)*	0 (±0.00)*
SIS	4.88 (±4.50)	3.73 (±4.64)	1.98 (±3.26)*
EAT-10	2.30 (±3.95)	1.13 (±3.45)*	0.39 (±1.36)*
Gerd-Q	2.28 (±3.64)	0.50 (±2.00)	0.24 (±1.26)*
RSI	9.33 (±9.53)	5.50 (±6.36)	2.79 (±3.89)*

*PO* postoperative

*p < 0.05 vs preoperative mean score

The statistical differences obtained by comparing the prevalence of score classes for each questionnaire are shown in Table 2. Before TT 32/40 (80%) of cases suffered voice symptoms. The percentage decreased not significantly 1 month after TT (32/40 vs 31/40 – *p* > 0.05) and decreased significantly 3 months after surgery (32/40 vs 25/40 – *p* < 0.05). Before TT 25/40 (62.5%) of patients complained of respiratory symptoms. The percentage decreased significantly at both postoperative controls (25/40 vs 7/40 and 0/40 – *p* < 0.05). Preoperatively the number of patients with swallowing symptoms was 38/40 (95%) and decreased after surgery reaching a statistical difference at both controls (38/40 vs 26/40 and 23/40 respectively – *p* < 0.05). Before TT 11/40 (27.5%) cases had the EAT-10 score above the cut-off for abnormal swallowing. The prevalence of cases with abnormal swallowing decreased significantly 1 and 3 months later (11/40 vs 4/40 and 1/40) (*p* < 0.05).

**Table 2:** The distribution of prevalence (%) basing on the score obtained by each questionnaire before and after thyroidectomy and the statistical differences obtained by comparing preoperative prevalence to each postoperative ones and early postoperative prevalence to the late one

Score	Pre-operative	1 month PO	3 months PO	*p*
VIS				
No dysphonia	8 (20%)	9 (22.5%)	5 (12.5%)	ns
Mild	22 (55%)	27 (67.5%)	30 (75%)	ns
Moderate	7 (17.5%)	7 (17.5%)	3 (7.5%)	ns
Severe	3 (7.5%)	7 (17.5%)	1 (2.5%)	ns
Very severe	0	0	1 (2.5%)	ns
mMRC				
0	15 (37.5%)	33 (82.5%)*	40 (100%)*	
Mild	12 (30%)	6 (15%)*	0*	
Moderate	3 (7.5%)	1 (2.5%)	0	ns
Severe	5 (12.5%)	0*	0*	
Very severe	5 (12.5%)	0*	0*	
SIS				
No dysphagia	2 (5%)	14 (35%)*	17 (42.5%)*	
Mild	25 (62.5%)	27 (67.5%)	20 (50%)	ns
Moderate	10 (25%)	7 (17.5%)	2 (5%)*§	
Severe	3 (7.5%)	1 (2.5%)	1 (2.5%)	ns
Very severe	0	1 (2.5%)	0	ns
EAT-10				
No dysphagia	29 (72.5%)	36 (90%)*	39 (97.5%)*	
Mild	9 (22.5%)	3 (7.5%)	1 (2.5%)*	
Moderate	2 (5%)	1 (2.5%)	0	ns
Severe	0	0	0	ns
Gerd-Q				
<8	36 (90%)	38 (95%)	39 (97.5%)	ns
>8	4 (10%)	2 (5%)	1 (2.5%)*	
RSI				
<15	32 (80%)	35 (87.5%)	39 (97.5%)*	
>15	8 (20%)	5 (12.5%)	1 (2.5%)*	

*PO* postoperative

*p < 0.05 vs pre-operative

After surgery the score of the items 0 “*Dyspnea only with strenuous exercise*”, 6 “*I have some difficulties for fluid swallowing*” and 9 “*I cough when I eat*” of mMRC, SIS and EAT-10 questionnaires respectively showed the greatest improvement. Before the TT the item 1 “*My voice is hoarse*” had the greatest score. On the other hand, after 1 month the score of item 2 “*My voice is breathy and weak*” of VIS showed the greatest worsening. Three month later the greatest improvement was reached in item 7 “*My voice changes during the day*” of the VIS score.

### Reflux symptoms questionnaires

Before TT 8/40 (20%) cases had the RSI score above the cut-off for airway reflux (Table 2). This prevalence significantly decreased 3 months later (8/40 vs 1/40) (*p* < 0.05). Preoperatively, 4/40 (10%) patients had a Gerd-Q score >8 suggestive of gastroesophageal reflux. After surgery, the prevalence of cases with high likelihood of having gastroesophageal reflux disease decreased significantly (4/40 vs 1/40) (*p* < 0.05).

The mean scores of pre- and postoperative RSI and Gerd-Q are shown in Table 1. Both the mean RSI and Gerd-Q scores improved significantly 3 month after thyroidectomy (*p* < 0.05).

Before surgery, the correlation between the SIS and Gerd-Q was weakly positive (*r* = 0.256) and the correlation between the score of SIS and RSI was moderately positive (*p* = 0.545). One month after surgery, the SIS showed a significant strongly positive correlation with the RSI (*r* = 0.748, *p* < 0.05) and a very weakly negative correlation with Gerd-Q (*r* = −0.100). The positive correlation between SIS and RSI became very strong (*r* = 0.813) 3 months after TT.

At the early control, the scores of items “*Heart burn, chest pain, indigestion, or stomach acid coming up*” and “*How often did you have stomach contents (liquid or food) moving upwards to your throat or mouth (regurgitation)?*” of the RSI and Gerd-Q questionnaires, respectively, improved the most. After 3 months the item “*Troublesome or annoying cough*” of the RSI and the item “*How often did you have stomach contents (liquid or food) moving upwards to your throat or mouth (regurgitation)?”* of the Gerd-Q improved the most.

### Thyroid weight related results

**Fig. 1:**
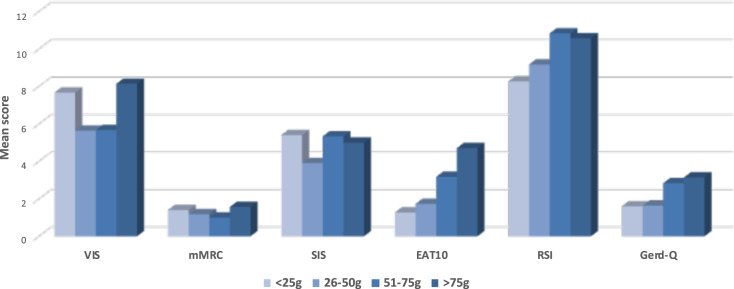
The preoperative mean scores stratified by groups of thyroid weight. *the EAT-10 mean score of >75 gr group. differ statistically if compared with the one of the <25 gr group

Before surgery, the scores of VIS, mMRC, SIS, RSI, and Gerd-Q did not differ between the groups of thyroid weight. On the other hand, the EAT-10 score of patients with the heaviest thyroid (group IV) was significantly higher than the score of group I (Fig. 1).

**Fig. 2:**
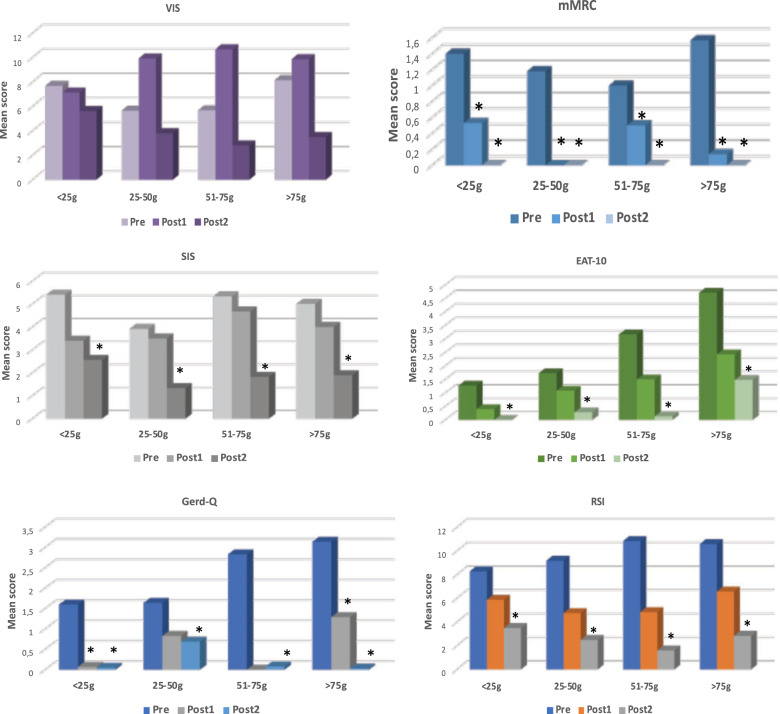
The pre- and post-operative mean scores for each questionnaire stratified by thyroid weight groups. *statistically different comparing with the preoperative mean score (*p* < 0.05)

As shown in Fig. 2, 1 month after surgery the VIS score increased in all thyroid weight groups except for group I, nevertheless the differences with the preoperative scores were not statistically significant (*p* > 0.05). After 3 months the VIS score improved not significantly (*p* > 0.05) in all groups of thyroid weight. The mean gain of VIS score was statistically significant improved in group IV if compared with other groups of thyroid weight (*p* < 0.05) (Table 3).

**Table 3:** Mean postoperative (PO) difference between the preoperative and postoperative scores (gain) and statistical significance (p) of the differences between the mean gains of each group of thyroid weight

PO gain	<25 gr	26–50 gr	51–75 gr	>75 gr
VIS				
*Early*	0.53	−4.28	−5	−1.71
Late	3	1.85	2.87	4.66*
mMRC				
Early	0.87	1.18	0.5	1.43*
Late	0	1.18	1	1.57*
SIS				
Early	2	0.41	0.67	1
Late	2.84	2.58	3.01	3.51*
EAT-10				
Early	0.87	0.64	1.67	2.29*
*L*ate	2	0.02	3.04	3.23*
RSI				
Early	2.40	4.42	6	4
Late	4	0.03	9.24	7.74
Gerd-Q				
Early	1.53	0.80	2.83	1.86
Late	3	0.11	2.75	3.11*

*p < 0.05

One month and 3 months after surgery the mean mMRC scores improved significantly in all thyroid weight groups (Fig. 2). Both postoperative mean gain of the mMRC score was statistically higher in group IV if compared with the other groups of thyroid weight (*p* < 0.05) (Table 3).

One month after surgery, SIS improved slightly in all weight groups, even if not statistically significant. Three months after TT the scores of all weight groups were statistically improved compared with preoperative (*p* < 0.05) sores (Fig. 2) and the mean gain of the SIS score was statistically higher in the group IV if compared with thyroid weight groups (*p* < 0.05) (Table 3).

The EAT-10 scores did not change significantly 1 month after surgery (*p* > 0.05) in all groups of thyroid weight. Three months after TT the score of all thyroid weight groups improved significantly (*p* < 0.05) (Fig. 2). Both postoperative mean gain of EAT-10 score was statistically higher in group IV if compared with the score of the other groups (*p* < 0.05) (Table 3).

As shown in Fig. 2, 1 month after surgery, the mean Gerd-Q score improved significantly (*p* < 0.05) in group I and IV and the mean RSI score did not change significantly in all thyroid weight groups. Three months after TT both Gerd-Q and RSI scores were statistically better than the preoperative ones in all thyroid weight groups. After 3 months the mean gain of Gerd-Q score was statistically higher in group IV if compared with the ones of the other groups of thyroid weight (*p* < 0.05) (Table 3).

**Fig. 3:**
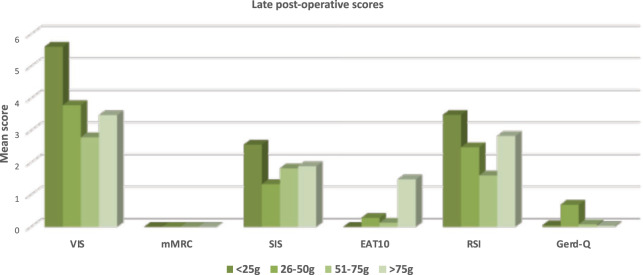
The late postoperative mean scores stratified by groups of thyroid weight. * the EAT-10 mean score of >75 gr group. differ statistically if compared with the one of the <25 gr group

Overall, at the late postoperative control, the scores of VIS, mMRC, SIS, RSI and Gerd-Q did not differ between the groups of thyroid weight (Fig. 3). The EAT-10 score of group IV was statistically higher than the one of the group I (*p* < 0.05).

## Discussion

In recent literature, a large interest has been paid to the occurrence of voice and swallowing impairments, which are related to thyroidectomy in the absence of laryngeal nerves injuries, and a smaller attention was addressed to the functional symptoms observed before the surgery. The results of this study, on the one hand, provide further confirmation that uncomplicated thyroidectomy may cause frequently temporary voice and swallowing changes and on the other hand, add interesting insight about preoperative functional complaints. The prevalence of preoperative self-reported voice and swallowing symptoms in our cohort was found to be 80% and about 95% respectively. A literature review reported that the prevalence of compressive symptoms in pre-operative thyroidectomy patients varied widely ranging from 11 to 88% [8, 11, 14, 21–23]. However, many of these studies differ concerning the methods, which makes comparisons between cohorts difficult. Certainly, it must be considered that our prevalence may be overestimated because of the cutoff was too low. On this regard, further researches will be necessary to review definitively the threshold of the questionnaires. Despite the aforementioned percentages, the preoperative overall mean scores were not significantly high. The trend of subjective perception of voice quality is similar to that described by us in the past [3, 17] as late improvement after initial worsening. In addition, we found the late subjective voice quality score was significantly improved than the preoperative score. It is likely that pathologic thyroid glands cause voice discomfort (physical, psychological), which, initially, worsens after surgery because of wound healing processes, typical for the early postoperative period, and then owing to stabilization of the wound and the removal of the pathologic gland, completely recovers over time. This last hypothesis is partially confirmed by voice symptom improvements observed in patients with the heaviest glands. Unlike dysphonia, dysphagia showed a tendency to recover more rapidly. Similarly, Greenbaltt et al. [23] described an abnormal pre-operative SWAL-QOL indicating the perception of impaired swallowing and imperfect quality of life, and after surgery a significant improvement in the most of SWAL-QOL domains. The validity of SIS was strengthened by the significant correlation with EAT-10. On this regard, we retain the SIS questionnaire more specific as demonstrated by the exact “*I have some difficulty swallowing fluids (*i.e.*, cough)*” of SIS respect to “*I cough when I eat”* of the EAT-10, which were the most frequent items reported for each questionnaire. Before the surgery, similarly to the voice related results, all patients complained swallowing symptoms, which was not correlated to thyroid weight. Nevertheless, thyroidectomy results in a significant improvement of voice and swallowing symptoms in patients with heaviest glands. Holler and Anderson [15] did not find preoperative significant correlation of VHI and SIS with thyroid volume on ultrasound and questioned the origin of these symptoms concerning LPR. However, they did not provide post-surgical data. In our cohort the preoperative endoscopic signs suggestive of LPR were found in 0.4% of patients, the mean scores of RSI and Gerd-Q were very low (far below the cutoff) and the number of patients who showed a clinically significant RSI and Gerd-Q scores were respectively 20 and 10%. Based on the overall data, we can affirm that before surgery, voice and swallowing complaints are possible regardless of reflux disease. After surgery, both reflux symptoms scores and percentages decreased, which suggests that TT in addition to voice and dysphagia improves also reflux symptoms. Nevertheless, to understand the role of the GERD, we described the subjective respiratory changes related to surgical thyroid pathology in uncomplicated thyroidectomy. Preoperatively more than half of the patients complained dyspnea and dyspnea severity did not differ in the thyroid weight groups. A mild dyspnea regressed after surgery particularly in cases with heavy thyroids. Dyspnea is traditionally expected in retrosternal goiter [24], carcinoma thyroid infiltrating recurrent laryngeal nerve, congestive cardiac failure in thyrotoxicosis or after surgery because of iatrogenic nerve injury [25]. Excluding these last conditions, the finding of dyspnea in our sample probably resulted from psychological reaction to the disease or direct compression of the respiratory organs by enlarged thyroid glands as proven by complete regression early after surgery [3, 5]. It is known that the items included in the RSI (the most used index) refer to dysphagia (i.e., “*difficulty swallowing food, liquids or pills*”), dysphonia (i.e., “*hoarseness or a problem with your voice*”) and respiratory abnormalities (“*breathing difficulties or choking episodes*”). In light of the overall functional outcome, we believe that the surgical disease of thyroid and the reflux disease share part of the symptoms. Particularly, SIS and RSI revealed a strong positive correlation before and after surgery suggesting the principal overlap.

In our past studies [3, 17], voice symptoms worsened in the early postsurgical assessment and returned as before when assessed during long term follow-up. Similarly results were observed for dysphagia except for patients who underwent videoassisted TT (symptoms ameliorated already 1 month after TT). Cusimano et al. [26] studied patients affected by reflux to explain the role of thyroidectomy in persistent neck symptoms. The authors speculated that surgery may worsen the reflux disease by reducing antireflux systems and suggests including reflux disease as cause for neck postoperative discomforts. In our cohort, we obtained a better and earlier functional outcome after traditional TT regardless of reflux disease. On this regard, it cannot be ruled out that the actual results come from improvement of surgical ability over time, which minimizes the surgical trauma of thyroidectomy in addition to the more favorable psychologic and emotional conditions of patients specifically counseled before TT.

Anyhow, we provided data about respiratory symptoms before and after TT offering an interesting input to further studies. On this regard, we believe that caregivers should consider respiratory abnormalities in the upper aerodigestive tract as specific discomforts commonly associated to the preoperative compressive syndrome. In addition, physicians should inform patients that dyspnea improves after uncomplicated TT in most cases.

The prevalence and severity of aerodigestive preoperative compressive symptoms (included respiratory ones) were globally reduced after surgery especially in cases with a heavy gland. This finding underscores the potential benefits of thyroidectomy in patients with benign goiter requiring an operation for compressive symptoms.

Overall, further studies are needed to optimize specificity of the psychometric test for surgical thyroid disease especially in order to define a reliable normal cutoff value and to reduce mis-overlap between the symptoms of thyroid and reflux diseases.

## Data Availability

We have full control of all primary data and they are agree to allow the journal to review their data if requested.

## Code availability (software application or custom code)

N/A (we did not use a software application).
